# Predictive Maintenance System to RUL Prediction of Li-Ion Batteries and Identify the Fault Type of Brushless DC Electric Motor from UAVs

**DOI:** 10.3390/s25154782

**Published:** 2025-08-03

**Authors:** Dragos Alexandru Andrioaia

**Affiliations:** The Department of Power Engineering and Computer Science, Faculty of Engineering, “Vasile Alecsandri” University of Bacau, 600115 Bacau, Romania; dragos.andrioaia@ub.ro

**Keywords:** PdM, UAV, sensors, IoT, BLDC fault identification, Li-ion RUL prediction

## Abstract

Unmanned Aerial Vehicles have started to be used more and more due to the benefits they bring. Failure of Unmanned Aerial Vehicle components may result in loss of control, which may cause property damage or personal injury. In order to increase the operational safety of the Unmanned Aerial Vehicle, the implementation of a Predictive Maintenance system using the Internet of Things is required. In this paper, the authors propose a new architecture of Predictive Maintenance system for Unmanned Aerial Vehicles that is able to identify the fault type of Brushless DC electric motor and determine the Remaining Useful Life of the Li-ion batteries. In order to create the Predictive Maintenance system within the Unmanned Aerial Vehicle, an architecture based on Fog Computing was proposed and Machine Learning was used to extract knowledge from the data. The proposed architecture was practically validated.

## 1. Introduction

Nowadays, humanity is enjoying the advantages of the fourth industrial revolution. Within “Industry 4.0”, the main objective of Predictive Maintenance (PdM) systems is the operation of equipment without failures [1,2,3,4,5].

In recent years, Unmanned Aerial Vehicles (UAVs) have started to be used more and more due to their unexploited potential. Thus, UAVs are used in fields such as: transport, leisure, search and rescue missions, environmental monitoring, agriculture, military, etc. The UAVs market is expected to grow to USD 100 billion by 2030 [6,7,8,9,10,11,12].

Along with the increase in the number of UAVs, the number of accidents has also increased exponentially. Failure of UAVs can lead to UAVs crash, loss of UAVs, injury to people and destruction of objects. Due to the rise in electrical capacity stored with the help of batteries, the number of UAVs that use electric motors is increasing. Most electrically powered UAVs use Brushless DC (BLDC) motors. A BLDC motor is a type of synchronous electric motor powered by direct current (DC), in which electronic commutation replaces the mechanical commutation typically found in brushed DC motors. The BLDC motor consists of a permanent magnet rotor and a stator with windings, where the switching of current in the stator windings is controlled by an electronic controller. For the switching of current in the stator of the BLDC motor, the electronic controller can use sensors to determine the rotor position (Hall sensors) [13,14,15,16].

BLDC motors have high power density. Due to the lack of brushes, their reliability is high [17,18,19,20]. For power, electrically powered UAVs use Li-ion batteries. With the increase in the number of charge–discharge cycles and due to the conditions of use, the capacity of Li-ion batteries decreases [21].

To increase the operational safety of the UAVs, it is necessary to implement a maintenance system. Strategies developed in the field of maintenance can be reactive and proactive [22,23].

Reactive maintenance strategies are also known as Run-to-Failure (R2F). R2F is one of the simplest and most traditional maintenance strategies used in industry. It involves making repairs after a piece of equipment or system has failed. Unlike planned repairs, emergency repairs have a much higher cost, being the most expensive [22,23]. Applying this maintenance strategy to UAVs can lead to failures that can result in: injury to people, loss of the UAV or material damage.

Proactive maintenance is a preventive maintenance strategy that aims to maintain the operational state of the equipment and involves repairing the equipment before it breaks down [23,24]. Proactive maintenance can be classified into: Preventive Maintenance (PvM) or PdM.

PvM involves applying a planned maintenance service before a fault occurs. This maintenance time can be based on: equipment operating time, calendar time or operating cycles [23,25,26]. Often, the application of PvM leads to the replacement and repair of parts and machines that do not actually need replacement or repair, which increases costs.

Due to the fact that service activities only occur when needed, PdM has become the desired industry standard in most markets. PdM is a maintenance strategy based on continuous monitoring of equipment operating parameters, using data analysis techniques and IoT sensors to prevent failure. By using PdM systems, equipment downtime is minimized, equipment maintenance costs are reduced and, consequently, production capacity is improved [27]. Thus, PdM can identify faults or estimate Remaining Useful Life (RUL) of equipment [1,25,28,29,30,31].

The developments of emerging technologies, such as Internet of Things (IoT), Big data (BD), Data Mining (DM), Internet of Services (IoS), Cyber-Physical Systems (CPS), and Cloud Computing (CC), make possible the implementation of PdM [32,33].

Based on sensor data that monitors the operation of equipment, PdM systems make component RUL predictions and identify faults. PdM systems whose decision-making system uses Machine Learning (ML) enable the identification of faults long before functional failure occurs [34,35,36,37,38]. Among the most used ML algorithms in PdM we mention: for *classification problems* Support Vector Machine (SVM), Random Forest, XGBoost and k-Nearest Neighbors (k-NN) and for *regression problems* Linear/Polynomial Regression, Random Forest Regressor, XGBoost Regressor/Gradient Boosting Machines. In PdM systems, regression is used to predict the RUL and classification is used to identify the occurrence of a fault (binary classification) or to classify the fault type (multiclass classification).

In developing PdM systems for UAVs using IoT, we can use CC-based architecture and Fog Computing (FC)-based architecture.

Data storage and processing in the cloud is cheaper, but has a number of disadvantages [39,40]. A CC-based architecture consists of the device layer and the cloud platform [41]. The devices have limited processing and storage power and are equipped with communication systems that are capable of transmitting data to the cloud. On the cloud layer, the data is stored and then processed. The cloud has a large data storage and processing power [42,43,44,45,46].

FC is an intermediate layer between the device layer and the cloud layer. In this way, the computing systems at the fog layer receive the data from the device layer and preprocess it, then the result is sent to the cloud where it is processed with greater computing power. FC differentiates between relevant and irrelevant data by sending only essential data to the cloud layer. FC was first proposed by Cisco to reduce the load on cloud datacenters by extending processing to the edge of the network [43,44,47,48,49].

FC and CC are different technologies; both cannot replace and change each other when it comes to their applicability. Generally, FC is used to process real-time sensitive information and CC is used to process non-time-based information.

Implementing PdM in industry also comes with a series of challenges such as: requiring experts in analysis and interpretation, high initial costs for implementation, risk of false positive or negative alerts, old equipment can be difficult to integrate into PdM systems, etc.

In this paper, the authors propose an architecture that PdM systems should have for UAVs, which can identify the fault type of BLDC electric motors and estimate the RUL of Li-ion batteries.

Since by using a cloud-based architecture, considerable bandwidth would be consumed for data transmission and the security of cloud data would be low, a FC-based architecture was used for the realization of the PdM system for the UAVs, which has consisting of three layers: the devices layer, the fog layer and the cloud layer. The first two layers, devices and fog, are part of the UAV, and the cloud layer is within the maintenance center.

The proposed PdM system architecture for UAVs has been practically validated.

### 1.1. State of Art

Regarding the architectures of the proposed PdM systems in the specialized literature, we can remember:

Wang Ke-Sheng proposed a general block diagram that any maintenance system should contain within the industry standard 4.0. The architecture of the proposed PdM system is composed of the following layers: 1. Sensory, deals with the acquisition of data from sensors; 2. Preprocessing and feature extraction, deals with signal processing and fault feature extraction; 3. Making maintenance decisions, dealing with data analysis to generate the prognosis; 4. Key Performance Indicators (KPI), *spider* charts are used to indicate component degradation; 5. Optimization, deals with the optimization of the maintenance program; 6. Feedback control and compensation, deals with error compensation [50].

Patrick Killeen, Bo Ding, Iluju Kiringa, and Tetîn Yeap, within the industrial standard 4.0, proposed the architecture of a PdM system for a bus fleet. The architecture is divided into three layers: 1. The perception layer, implemented on each bus, contains sensors, limited storage media, communication devices; 2. The middleware layer, characterized by larger storage space, ML, provides the interface with the nodes of the perception layer; 3. The application layer, which displays the results, user management, and platform administration. The data is taken from the CAN interface of the buses. Application layer communication is done via MQTT protocol [51].

Zhe Li, Yi Wang, and Ke-Shengpropun Wang proposed a block diagram of a PdM, which can be used to diagnose malfunctions of machine tool centers. The block diagram consists of five layers: 1. The sensors selection and data acquisition layer, selects the appropriate sensors and collects the data; 2. Data preprocessing layer, deals with data storage and preprocessing; 3. Data extraction layer, deals with extracting knowledge from data; 4. The decision and support layer, allows viewing the result provided by the DM; 5. Maintenance strategy implementation layer, maintenance will be implemented after the decision makers choose the maintenance strategy [52].

Erim Sezer, David Romero, Federico Guedea, Marco Macchi, and Christos Emmanouilidis propose a PdM that can identify the worn tools of a turning machining center, based on the analysis of data from temperature and vibration sensors of the cutting tool. It uses a cloud-centric architecture where a Raspberry Pi handles the acquisition and transmission of sensor data to the cloud where it is analyzed. The PdM system uses ML to make predictions. Temperatures and vibrations are labeled according to the quality of the parts resulting from the cutting process [53].

Dimitris Mourtzis, John Angelopoulos, and Nikos Panopoulos, propose a PdM for refrigerators that aims to reduce downtime by 20% and reduce maintenance costs by 10%. The architecture used is cloud-centric. The devices monitor the operation of the refrigerators via pressure (compressor), temperature and humidity sensors and then transmit the data to the database located in the cloud via XBee modules. The data is then analyzed using ML to identify faults and predict RUL [54].

Tung Tran Van, Ian Chan, Sagunthala Parthasarathi, Choon Pio Lim, and Yong Quan Chua propose a PdM system to diagnose component failures of a plastic welding machine. The device at the edge of the network takes data from the machine’s sensors as well as the PLC used to control the machine and transmits it to a local server, where it is analyzed. Faults are identified through ML algorithms. The data is displayed via a web interface [55].

Irene Niyonambaza, Marco Zennaro, and Alfred Uwitonze propose a PdM system using IoT that allows for the prediction of mechanical equipment failures in hospitals in Rwanda, which aims to reduce downtime for medical staff. Within the system architecture, IoT devices transmit equipment monitoring data to a database hosted by a local server, to which an application is connected that allows for the prediction of failures. Within the experimental results obtained, for the prediction of component failures of a steam sterilization generator, the authors used Long Short-Term Memory (LSTM) neural networks, resulting in a prediction accuracy of 90% and 96% for components [56].

Muhammad Umair Hassan, Ole-Martin Hagen Steinnes, Eirik Gribbestad Gustafsson, Sivert Løken, and Ibrahim A. Hameed propose a PdM system that uses Deep Learning (DL) to classify roads in Norway according to the degree of deterioration. To develop the proposed PdM system, the authors used images from a video camera that filmed the roads as input data. Various variants of Convolutional Neural Networks (CNNs) were used to classify the road condition. For vertical cracks, one of the analyzed models obtained 50% for average precision and 73% for F1-score [57].

Eyup Cinar, Eyup Cinar, and Inci Saricicek propose an architecture of PdM systems for manufacturing plants. In the proposed architecture, the data from equipment monitoring is transmitted via the MQTT protocol to the server where it is stored via a database. Based on the data stored in the database, the PdM application, using ML and DL techniques, identifies incipient faults and estimates the RUL of the equipment. The proposed PdM system was validated on an electric motor test bench [27].

Ruiqi Tian, Santiago Gomez-Rosero, and Miriam A.M. Capretz propose a PdM system for Heating, Ventilation, and Air-Conditioning (HVAC) systems with the aim of minimizing the environmental footprint. Based on data such as energy consumption and temperature of the HVAC unit, two fault types, a clogged air filter and a leak in the air ducts, are identified using Health Prognostics Classification with Autoencoders (HPC-AE). The authors also compare the performance of HPC-AE with the performance of other autoencoders and find that HPC-AE shows an increase of 5.7% and 2.1% in the F1 score in identifying the two fault types [58].

Mubashar Iqbal, Sabah Suhail, Raimundas Matulevičius, Faiz Ali Shah, Saif Ur Rehman Malik, and Kieran McLaughlin propose a PdM system for automobiles, with the aim of identifying faults before they occur. The proposed PdM system integrates the technologies: Digital Twin, ML and Blockchain. Digital Twin provides real-time monitoring of vehicle operating conditions, ML facilitates data-driven predictions for predictive vehicle maintenance, while blockchain (nu it) is using for data integrity and traceability within physical and twin environments [59].

Agussalim Syamsuddin, Andrew Cahyo Adhi, Amie Kusumawardhani, Toni Prahasto, and Achmad Widodo propose a PdM system for a photovoltaic (PV) panel park, which allows for the detection, diagnosis and forecasting of anomalies. The PdM system uses real-time data acquired through the SCADA system that describes the operation of the PV panels. The PdM system uses DL to make predictions. The sensor parameters involved in the development of the system are ambient temperature, humidity, solar irradiation, wind direction and speed, while the estimated parameter is active power. Historical data on the variation of these parameters were used to create a DL model using a Long Short-Term Memory Autoencoder (LSTM-AE) algorithm. From the experimental results, it is found that the model created using the LSTM-AE algorithm has parameters that express the performance of MSE = 10.95, RMSE = 10.95, MAE = 3.30 in anomaly prediction [60].

Analyzing research in the specialized literature, it was found that specialized works dealing with the development of PdM systems for UAVs are limited, almost non-existent. Most authors focus on developing PdM systems for industrial equipment.

### 1.2. Structure of the Work

The paper is organized as follows:

Section 1 presents the need to implement a PdM system that uses IoT within the UAV, as well as the main concerns of researchers from the specialized literature regarding PdM systems architecture from various fields.

Section 2 presents the architecture of the proposed PdM system for UAVs, as well as its implementation.

Section 3 presents how to develop ML models that were used in the experimental stand, for predicting the RUL of Li-ion batteries and identifying the fault type of BLDC motors in the PdM component of UAVs.

Section 4 presents the obtained experimental results, which validate the architecture of the proposed PdM system for UAVs.

Section 5 presents the conclusions of this study.

## 2. Architecture of the Proposed PdM System

PdM involves continuous monitoring of equipment to anticipate equipment failures before they occur. The main goal of PdM is to keep equipment functional. Among the main benefits of implementing PdM in UAVs are: reduced downtime, optimized maintenance costs, increased lifespan, and improved safety.

**Figure 1:**
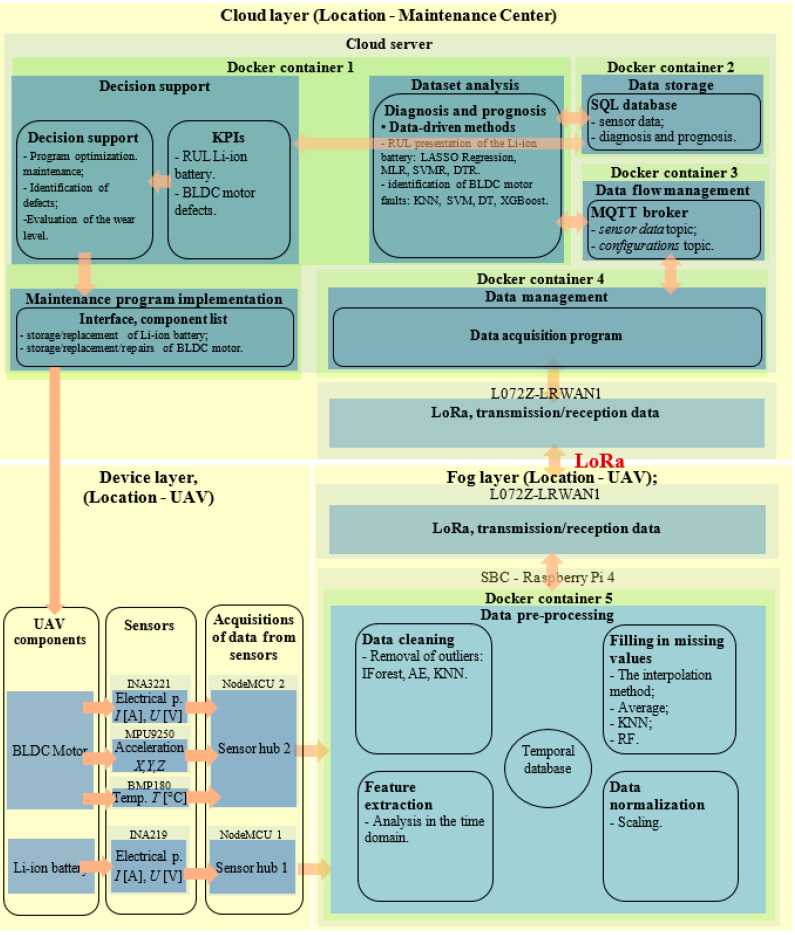
Block diagram of proposed PdM system for UAVs.

The proposed PdM system allows to identify the fault type of the BLDC electric motor and estimates the RUL of Li-ion batteries from UAVs. To minimize the amount of data sent to the cloud, an FC-based architecture was used. A block diagram of the proposed PdM system for UAVs can be seen in Figure 1.

It consists of three layers: the devices layer, the fog layer, and the cloud layer. The devices layer deals with sensor data acquisition and transmission, the fog layer deals with data preprocessing, and the cloud layer deals with data processing, storage, and display.

**Figure 2:**
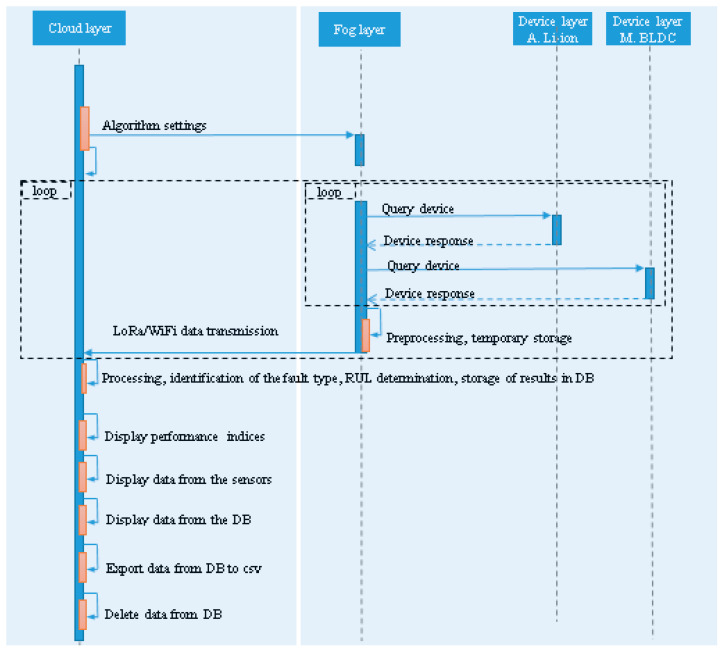
UML sequence diagram of proposed application.

The UML sequence diagram of the software application can be viewed in Figure 2. The sequence diagram shows how the processes work together and in what order.

On the device layer (Figure 1 and Figure 2) we have two devices. The first device (Figure 2, *Device layer M. BLDC*), monitors the parameters of the BLDC motor and the second device (Figure 2, *Device layer A. Li-ion*), monitors the parameters of the Li-ion battery.

The data is transmitted from the device layer to the fog layer (Figure 1 and Figure 2), where data is temporarily stored and then preprocessed. The fog-layer SBC continuously polls each device to obtain data from sensors, Figure 2.

Finally, only relevant data is transmitted from the fog layer to the cloud layer (Figure 1 and Figure 2). The data is stored on the cloud layer, then through ML models, the fault type of the BLDC motors is identified and the RUL of the Li-ion batteries is estimated.

The resulting data is displayed via web interfaces. The performance indices are displayed using the display performance indices interface (Figure 2). The display sensor data interface (Figure 2) allows to view the data from the sensors in real time. The *View data from BD*, *Delete data from BD*, *Export data from BD to csv* interfaces (Figure 2) allow display, export, and delete data from the database. The user can set various combinations of algorithms via the *Data settings* interface (Figure 2).

The composition and functioning of each layer are explained in more detail below.

### 2.1. The Devices Layer

On the device layer Figure 1, are IoT devices that take information from sensors. Each IoT device (sensor hub) receives information from sensors that monitor a certain component. Devices on the device layer will transfer data via the wired connection to the fog layer.

Within the block diagram in Figure 1, to determine the RUL of Li-ion batteries, NodeMCU 1, monitors Li-ion battery discharge *U [V]* and *I [A]* through the sensory module INA219 and determines the discharged capacity in real time. Sensory module INA219 Figure 1, communicates with the NodeMCU 1 development board via the I2C protocol. As the discharge load of the Li-ion battery, a BLDC motor A2208 was used.

In order to identify the fault type of the BLDC motor, operating parameters of the BLDC motor were monitored with the help of the NodeMCU 2 development board. The NodeMCU 2 development board reads: *U [V]* and *I [A]* which closes through the ESC of the BLDC electric motor by using the INA3221 sensory module, the accelerations *AccX [g], AccY [g], AccZ [g]* of the BLDC electric motor using the MPU-9250 sensory module and *T [C]* of operation of the BLDC electric motor using the BMP180 sensor module. The INA3221, MPU-9250, and BMP180 communicate with the NodeMCU 2 development board via the I2C protocol. The Arduino IDE 1.X was used to create the software applications for the two NodeMCUs.

### 2.2. The Fog Layer

The Raspberry Pi 4 SBC (Figure 1) interrogates via the UART protocol, the NodeMCU 1 development board to extract the *I [A], U [V],* and *C [mA/H]* operating parameters of the Li-ion battery, then queries the NodeMCU 2 development board to extract the operating parameters *I [A], U [V], AccX [g], AccY [g], AccZ [g],* and *T [°C]* of the BLDC electric motor.

On the fog layer, the data is preprocessed, thus reducing the amount of data transmitted to the cloud layer. Thus, on the fog layer, the data is cleaned, completed, transformed and normalized. During the cleaning process, outlier values are identified. Data transformation involves data analysis with the help of statistical indicators in the time domain. By normalizing, the data is scaled to the range 0–1. The Raspberry Pi 4 SBC preprocesses the data, stores them temporarily, and transmits the preprocessing result to the cloud.

Software applications on the fog layer and cloud layer contain a micro-services-based architecture. The software application on the fog layer runs in the Doker 5 container. The *Python 3.6* language was used to create the software application. Among the libraries used to create the software application on the fog layer, we can mention: *pySerial* for creating serial transmission; pandas and *numpy* for data processing; *PyOD* and *scikit-learn* for algorithms for filling in missing values and identifying outliers, etc.

From the fog layer to the cloud layer, data is transmitted via the LoRA (Long Range) protocol (Figure 1, L072Z-LRWAN1), thus is a continuous link between the fog layer and the cloud layer. LoRa is a narrowband wireless communication protocol, mainly used for LPWAN networks. Unlike other wireless technologies such as ZigBee or BLE, LoRa allows long-distance communication (15 km rural, 2–5 km urban) with low power consumption, making it ideal for IoT applications, where devices are dispersed over large areas and operate on batteries for long periods of time. LoRa uses CSS modulation, which provides resistance to interference and allows communication over long distances, even in noisy or urban environments [61,62,63].

The components of the UAV are continuously monitored, thus allowing the operator to see the status of the assets in real time.

### 2.3. The Cloud Layer

Communication at the application layer is done via MQTT protocol. The MQTT protocol is an application-layer messaging protocol based on the TCP/IP transfer. MQTT has a publisher–subscriber model and has a client-server architecture. Clients publish messages on specific topics and subscribers receive them. The MQTT broker deals with mediating the connection between publishers and subscribers. The MQTT broker is located on the server [64,65].

For cloud-layer implementation, the Mosquitto broker was used, which uses the MQTT protocol [64]. Mosquitto was installed within the *Doker 3* container within the cloud layer, Figure 1. [66]. Within the block diagram from Figure 1, the editor contained in the *Data acquisition program* block, located in the *Doker 4* container, receives data from the sensors through the LoRa protocol and sends the data to the *Doker 2* container to store it in the database. The data transmitted on the cloud layer are stored in the *sql* database, hosted through XAMPP application, the *Data storage* block from Figure 1.

The *Diagnostic and prognostic* block (Figure 1) located in the *Doker 1* container, based on data extracted from the *sql* database, extracts the RUL of the Li-ion battery, and identifies the fault type of the BLDC motor. Data-driven methods using ML were used for RUL extraction of the Li-ion battery and identifying the fault type of the BLDC motor.

**Figure 3:**
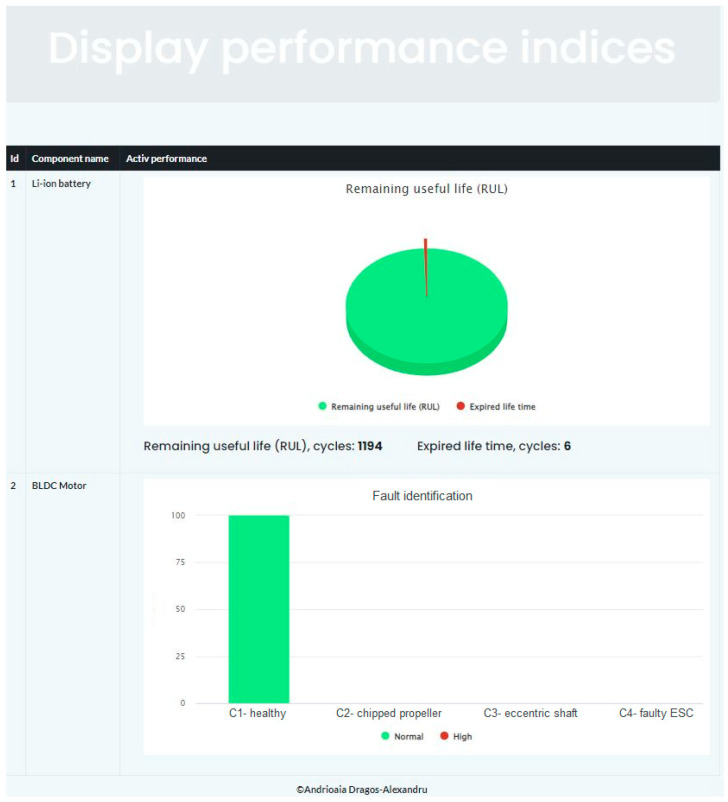
Web interface, *Display performance indices*.

Maintenance decisions are displayed via the *Decision Support block* (Figure 1). The *Display performance indices* interface in Figure 3 allows displaying the RUL of Li-ion batteries, as well as displaying BLDC motor faults.

The model created to identify the fault type of BLDC motors from UAVs can identify the operation under the following classes: *C1—healthy, C2—chipped propeller, C3—eccentric shaft and C4—faulty ESC.*

The *Maintenance program implementation* block (Figure 1) allows the implementation of the maintenance program after the decisions to keep/repair/replace equipment have been made.

When creating the software application on the cloud layer, the *Python* language was used. Flask extension was used to realize web user interface, *flask_mqtt* is an extension used to implement MQTT client.

## 3. Creating ML Models That Allow Prediction of the RUL of Li-Ion Batteries and Identification of the Fault Type of BLDC Motors in the PdM Component of UAVs

Among the most common failures of BLDC motors in UAVs, we can mention propeller failures, motor shaft eccentricity failures, as well as other electrical failures. Electrical failures can be caused by loss of insulation of the BLDC motor windings or by ESC failures.

A data-driven method using supervised ML was used to identify the fault type in BLDC motors from UAVs. The method uses historical data resulting from monitoring the operation of BLDC motors from UAVs under the influence of different classes of faults. The historical data was obtained by monitoring, using temperature, current, voltage, and vibration sensors, the operation of BLDC motors from UAVs under the influence of classes such as, *C1—healthy, C2—chipped propeller, C3—eccentric shaft, and C4- faulty ESC.* In the case of the faulty ESC class, the operation of the BLDC motor was monitored when a MOSFET transistor such as FDD8896 from the ESC component is faulty.

Thus, the health of the motor is expressed through parameters such as temperature, current, voltage, and vibration. During the operation of the BLDC motor when one of the analyzed faults occurs, the values of these parameters will change. The block diagram in Figure 3 illustrates the flow used to identify the fault type of BLDC motors.

**Figure 4:**
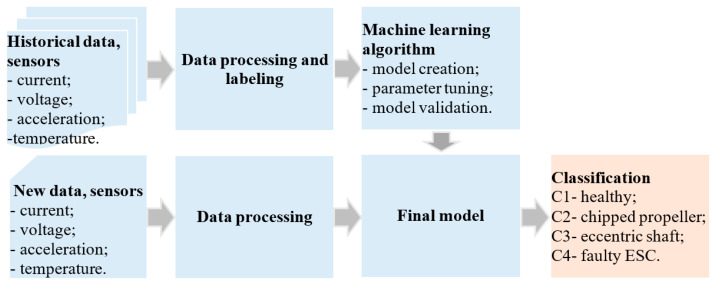
Diagram of process used to identify fault type in BLDC motors.

In the block diagram in Figure 4, the final model is created based on the data from monitoring the BLDC motor when it operates under the influence of classes such as *C1—healthy, C2—chipped propeller, C3—eccentric shaft, and C4—faulty ESC.* Before training the model, the historical data was processed and labeled. Data processing involves the operations of eliminating outlier values, filling in missing values and normalization.

In order to identify the algorithm that has the best performance in the classification of the fault type of BLDC electric motor, the performances of several algorithms were compared, as: K-Nearest Neighbors (K-NN), Support Vector Machine (SVM), Decision Tree (DT), and eXtreme Gradient Boosting (XGBoost). The performances of ML algorithms used for classifying BLDC motor faults in UAVs were evaluated through indicators: *ACC, PR_macro avg,_ Recall_macro avg,_ F1_macro avg._* The data used to train the models created using the four algorithms as well as the performance of the models are described in more detail in Section 5. Of the four models, the model that performed best in identify the fault type in BLDC motors was used.

After the final model was chosen, it was saved in a file with the .pkl extension and then uploaded to the cloud to make predictions. When new data from real-time sensors is applied to the model, it can identify the operation of the motor under the influence of one of the four analyzed classes.

Pickle (.pkl) is a standard Python module that allows serialization and deserialization of Python objects, which is often used to save trained models.

The degradation of Li-ion batteries is influenced by operating conditions as well as battery chemistry. Rapid discharges lead to a sudden decrease in the storage capacity of Li-ion batteries [67,68,69,70].

Model-based, data-based, and hybrid methods can be used to predict the RUL of Li-ion batteries. Model-based methods require the development of a physical model describing the aging process to predict the RUL of Li-ion batteries. Data-based methods do not require the development of a physical model describing the dynamics of the aging process of Li-ion batteries, they use supervised ML to predict the RUL of Li-ion batteries. Hybrid methods combine the advantages of both methods [21,70,71,72,73,74,75].

A data-driven method using supervised ML to make predictions was used to predict the RUL of Li-ion batteries in UAVs. The method uses historical data from sensors that captures the degradation process of Li-ion batteries under the influence of repeated charge–discharge cycles. For this study, historical data from monitoring the degradation of three Li-ion batteries in UAVs were used.

**Figure 5:**
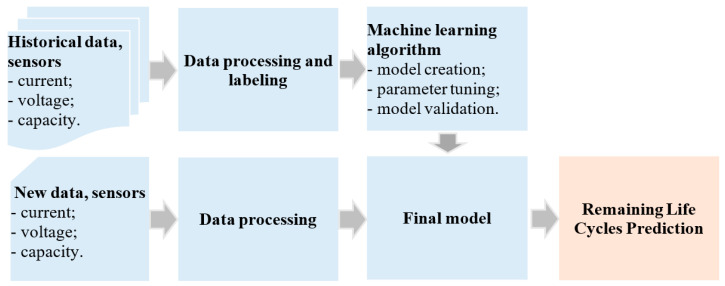
Diagram of process used for real-time RUL prediction.

The information flow used to predict the remaining life cycles of Li-ion batteries in UAVs is presented in Figure 5.

From the block diagram in Figure 5, it can be seen that the final model is created using historical data from current and voltage sensors. Before training the model, the historical data were preprocessed and labeled. During the data processing stage, outlier values were removed, missing values were filled in, and finally the data were normalized.

In order to identify the algorithm that has the best accuracy in predicting the RUL of Li-ion batteries, the performances of the algorithms were compared: Least Absolute Shrinkage and Selection Operator Regression (LASSO Regression), Multiple Linear Regression (MLR), Support Vector Machines Regression (SVMR), and Decision Tree Regression (DTR). The performances of ML algorithms used for predicting the RUL of Li-ion batteries from UAVs were evaluated through regression indicators: MSE, RMSE, MAE, and R2. The data used to create the models using the four algorithms as well as the performance of the models are presented in more detail in Section 5. Of the four models analyzed, the model that can identify the RUL of LI-ion batteries with better accuracy was used.

After the final model was selected, it was saved in a file with the. pkl extension and then uploaded to the cloud to make predictions. When new data from real-time sensors are applied to the model, it can estimate the RUL of Li-ion batteries.

## 4. The Experimental Stand

**Figure 6:**
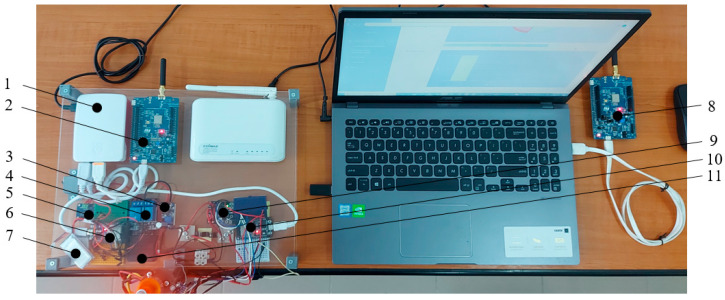
Experimental stand, top view: 1—Raspberry Pi 4; 2—LoRa module 1, L072Z-LRWAN1; 3—voltage booster module MT3607; 4—switching module, two relays; 5—INA2019 sensory circuit; 6—NodeMCU 1 development board; 7—Li-ion battery; 8—LoRa module 2, L072Z-LRWAN1; 9—INA3221 sensor circuit; 10—NodeMCU 2 development board; 11—Li-ion battery charging module MT3608.

**Figure 7:**
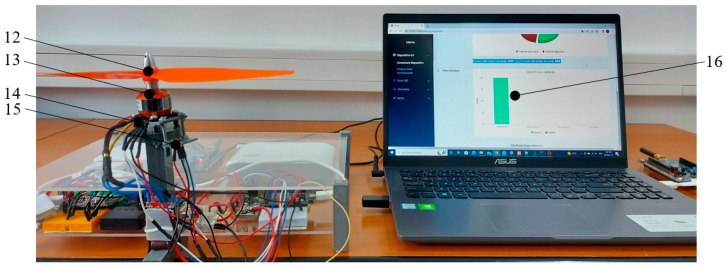
Experimental stand, front view: 12—propeller; 13—BLDC motor A2208; 14—BMP180 temperature sensor circuit; 15—vibration sensor circuit MPU9250; 16—laptop.

The experimental stand in which the block diagram in Figure 1 was implemented is shown in Figure 6 and Figure 7.

Within the experimental stand, the Li-ion battery (Figure 6 and Figure 7) is charged using module—11 in Figure 6. After the Li-ion battery is charged, the relay—4 from Figure 6—switches from the charging position to the discharging position. A BLDC electric motor (13 in Figure 7) was used as the discharge load for the Li-ion battery. Since the supply voltage of the BLDC electric motor driver is at least 7 [V], a voltage booster module MT3607 (3 in Figure 6) was used to obtain this voltage from the Li-ion battery (3.7 [V]).

Data from Li-ion battery monitoring (7 in Figure 6) obtained from the INA2019 sensory module that monitors *I* [A], *U* [V] (5 in Figure 6) are transmitted by NodeMCU 1 (6 in Figure 6), to the Raspberry Pi 4 SGB module (1 in Figure 6) via the USB port.

Data from BLDC electric motor monitoring (13 in Figure 7) such as *I* [A], *U* [V] using the INA3221 sensory module (9 in Figure 6), *T* [C] using the BMP180 sensor module (14 in Figure 7) and acceleration *AccX* [g], *AccY* [g], *AccZ* [g] using the MPU9250 sensor module (15 in Figure 7) are transmitted by the NodeMCU 2 development board (10 in Figure 6) to the Raspberry Pi 4 SGB module (1 in Figure 6).

The Raspberry Pi 4 SGB module (1 Figure 6) preprocesses the data and transmits the preprocessing result via the LoRa L072Z-LRWAN1 module (2 in Figure 6) to the laptop (16 in Figure 7), which receives them via of the LoRa module, L072Z-LRWAN2 (8 in Figure 6).

For this study, the BLDC A2208 motor was used, which has the following characteristics: KV (RPM/Volt) = 1100 [KV]; Nominal current = 10–20 A (depending on the propeller); Impedance = 225 [mΩ]; Weight = ~50 [g]; Number of poles = 12N14P (12 windings, 14 magnets). To transmit the data from the fog layer to the cloud layer, a LoRa peer-to-peer connection was implemented. LoRa allows data transmission up to distances of 15 [km] with low power. In a data packet, we can transmit between 2–255 bytes with a transmission speed of 50 [Kbps] [76]. The laptop simulates the cloud layer.

## 5. Results and Discussions

The PdM system developed for UAVs allows for the identification of the fault type in BLDC motors and the estimation of the RUL of the Li-ion battery.

By analyzing the results obtained, it is found that when we use LoRa for data transmission, the communication speed is much lower compared to data transmission using other types of transmission such as Wi-Fi, BLE, etc. Instead, LoRa allows the transmission of data over long distances.

The training data used to train the models that allows the identification the fault type of the BLDC electric motor comes from the PdM system shown in Figure 1, Figure 6 and Figure 7. These data were collected by monitoring the operation of a BLDC motor using temperature, current, voltage, and vibration sensors, under the influence of the following classes: *C1—healthy, C2—chipped propeller, C3—eccentric shaft, and C4—faulty ESC* (defective MOSFET transistor, FDD8896). To eliminate the influence of the external temperature on the motor temperature measurement, the ambient temperature measured with an external sensor was extracted from the temperature measured by the BMP180 motor sensor. The temperature difference between the two sensors was used to train the ML models. The database used to train the ML models contains data collected by monitoring the operation of the BLDC motor for 2 h, 30 min for each class, at a sampling rate of 10 Hz. The data was manually labeled.

The training data used to train the ML models that allows estimating the RUL of Li-ion batteries were obtained from the PdM system shown in Figure 1, Figure 6 and Figure 7 by monitoring the charge–discharge cycles of three Li-ion batteries, using data from current and voltage sensors. The database used to train the ML models contains data from monitoring 1200 charge–discharge cycles of three Li-ion batteries. After 1200 charge–discharge cycles, the capacity of the Li-ion batteries decreased to 30% of the initial capacity. The charging and discharging process of each battery lasted 21 days. The monitoring was performed at a sampling rate of 1 Hz.

The historical data used to train the ML models was split into 70% training and 30% testing data. The models were trained using the training data and the performance of the models was verified on the test data.

The performances of the models were analyzed based on the main performance indicators used in the specialized literature. Finally, the models were implemented within the proposed PdM system.

In Table 1, the performances of the algorithms in classifying the fault type (classifying the 4 faults type) of the BLDC electric motor are presented.

In order to demonstrate the effectiveness of models in classifying the fault type of BLDC electric motor, the performance of KNN, SVM, DT, and XGBoost algorithms in classifying the four operating conditions of BLDC electric motors was studied. The XGBoost classifier was able to identify the fault type of the BLDC electric motor most correctly. By modifying the symptoms of a fault by changing the chipped surface of the propeller, it was found that the performance of methods to classify this fault decreased. The XGBoost classifier was used to classify the fault type of BLDC electric motor, Figure 1.

In Table 2, the performances of the algorithms (LASSO Regression, MLR, SVMR, and DTR) in the RUL prediction of Li-ion batteries are presented.

Among the four algorithms used, LASSO Regression, MLR, SVMR, and DTR, the SVMR and DTR algorithms performed best in estimating the remaining life cycles of Li-ion batteries. For the RUL prediction of the Li-ion battery, the DTR algorithm was used, Figure 1. Following the RUL estimate, we can identify the number of charge and discharge cycles remaining in the life of the LI-ion battery.

## 6. Conclusions

The use of IoT, ML, DL, IoS, and PdM technology within the industry will bring great benefits. By being able to anticipate failures in advance, it helps companies to increase productivity, minimize maintenance costs, and improve the safety of the work environment.

In this work, the architecture of a PdM system based on FC that can be used for classifying the fault type of BLDC electric motor and predicting the RUL of Li-ion batteries from UAVs, was presented. Thus, the personnel in the maintenance service depending on the result given by the PdM system can replace the defective components in order to keep the asset in operation.

Data preprocessing on the fog layer allows reducing the amount of data that must be transferred to the cloud layer.

The prediction of faults as well as the RUL of the components can be done by continuously monitoring parameters that can inform us about the state of health as well as about the trend of evolution. To identify the fault type of the BLDC electric motor, the vibrations, the temperature, as well as the supply voltage and current were monitored, and to estimate the RUL of the Li-ion batteries, the voltage and current were monitored. In Figure 3, the BLDC motor operates under the influence of class *C1—healthy*. If the motor operates under the influence of classes: *C2—chipped propeller*, the propeller should be replaced; *C3—eccentric shaft*, the BLDC motor should be repaired or replaced; *C4—faulty ESC*, in this case the ESC should be repaired or replaced. The stability of the UAV when operating under the influence of a BLDC motor failure, depends on the number of BLDC motors. The higher the number of BLDC motors, the more tolerant the UAV is to a BLDC motors failure.

LoRa communication allows data transfer over long distances but at the cost of a low transfer rate. Thus, data transmission via LoRa allows continuous monitoring of the asset.

To increase the reliability of the UAV, other components that have a high probability of failure can be analyzed.

The effectiveness of the PdM system architecture proposed for UAVs was demonstrated through the obtained experimental results.

## Figures and Tables

**Table 1:** Performance of algorithms used to classify fault type of BLDC electric motor.

Performance Indicator	*KNN*	*SVM*	DT	XGBoost
*ACC*	0.932	0.966	0.979	0.982
*PR_macro avg_*	0.934	0.968	0.980	0.982
*Recall_macro avg_*	0.932	0.966	0.979	0.982
*F1_macro avg_*	0.932	0.966	0.979	0.982

**Table 2:** Performance of models used to predict RUL of Li-ion batteries.

Performance Indicator	*LASSO Regression*	*MLR*	*SVMR*	*DTR*
*MAE*	67.399	67.210	18.877	8.993
*MSE*	7615.849	7614.101	1007.653	154.687
*RMSE*	87.268	87.258	31.743	12.437
*R^2^ Score*	0.938	0.938	0.992	0.998

## Data Availability

All data are presented in the main text.

## Abbreviations

The following abbreviations are used in this manuscript:

BLDC	Brushless DC
CC	Cloud Computing
CPS	Cyber-Physical Systems
DL	Deep Learning
DM	Data Mining
DT	Decision Trees
DTR	Decision Tree Regression
FC	Fog Computing
HVAC	Heating, Ventilation, and Air-Conditioning
IoS	Internet of Services
IoT	Internet of Things
K-NN	K-Nearest Neighbor
LASSO	Least Absolute Shrinkage and Selection Operator
LSTM	Long Short-Term Memory
LSTM-AE	Long Short-Term Memory Autoencoder
ML	Machine Learning
MLR	Multiple Linear Regression
PdM	Predictive Maintenance
PV	Photovoltaic
PvM	Preventive Maintenance
R2F	Run-to-Failure
RUL	Remaining Useful Life
SBC	Single-Board Computer
SVM	Support Vector Machine
SVMR	Support Vector Machines Regression
UAV	Unmanned Aerial Vehicle
XGBoost	eXtreme Gradient Boosting
